# Coated Bipolar Membranes with Improved Forward Bias Performance for Energy Harvesting from Salt-Contaminated Acid and Base

**DOI:** 10.1021/acselectrochem.5c00052

**Published:** 2025-05-14

**Authors:** Nadia Boulif, Kitty Nijmeijer, Zandrie Borneman

**Affiliations:** Membrane Materials and Processes, Department of Chemical Engineering and Chemistry, 3169 Eindhoven University of Technology, P.O. Box 513, 5600 MB Eindhoven, The Netherlands

**Keywords:** bipolar membrane, forward bias, ionic blockade, electrochemical impedance spectroscopy, reverse electrodialysis, polymeric coating

## Abstract

Background sodium chloride in hydrochloric acid and sodium hydroxide solutions leads to large overpotentials when a bipolar membrane (BPM) is operated under forward bias (FB). Under FB polarization, the accumulation of salt ions at the junction hinders the transport of H^+^ and OH^–^ ions, thus increasing the mass transport resistance and lowering the water recombination rate. The “ionic blockade” phenomenon is mainly observed if the base is contaminated with Cl^–^ ions due to the poor OH^–^/Cl^–^ selectivity of the BPM’s anion exchange layer (AEL). This shortcoming is successfully reduced by modifying the AEL with a sub-micrometer thick poly­(benzimidazole) (PBI) coating. Ionic crosslinking between the AEL and PBI leads to a denser interface that enhances the size exclusion of Cl^–^ ions. Furthermore, the negative charges of deprotonated benzimidazole units at the basic operating conditions contribute to the Donnan exclusion of Cl^–^ ions, while the OH^–^ ions can still hop between the alkaline-doped free volumes of the PBI film. The enhanced OH^–^/Cl^–^ selectivity prevents the accumulation of Cl^–^ ions at the junction and leads to lower overpotentials during the forward bias operation of BPMs in salt-contaminated acid and base. As a result, the PBI-coated BPM has a peak power density 1.6 times higher than that of an uncoated BPM when harvesting electrical energy from a pH gradient. The BPM modification also benefits flow battery applications, as the calculated BPM voltaic efficiency at 100 A/m^2^ (dis)­charge current density is increased from −3.7% to 57% with the addition of the PBI coating.

## Graphical abstract

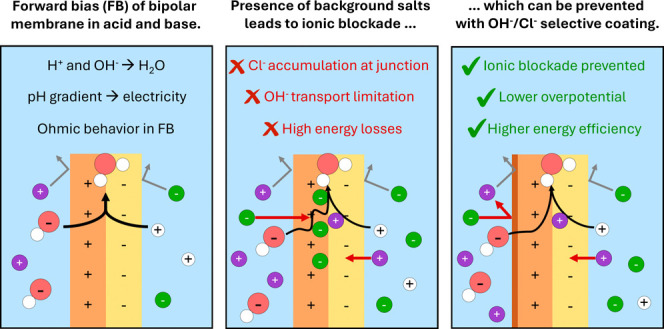

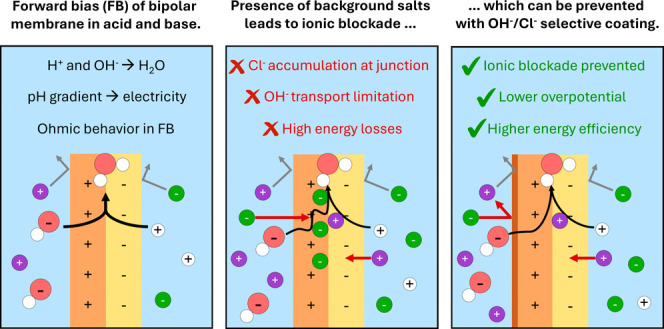

## Introduction

In the changing energy landscape, electrochemical systems and devices for energy storage and harvesting are of foremost importance.^1–3^ In this context, bipolar membranes (BPMs) play a key role, and their contribution to the electrochemical field has shown a strong increase in recent years.^4–6^ In reverse bias (RB), BPMs convert electrical energy into chemical potential energy via the dissociation of water in its respective protons and hydroxide ions at the BPM junction. Reverse bias operation is well known for its application in, e.g., the production of acids and bases, CO_2_ capture, ammonia recovery, and wastewater treatment.^5^ In forward bias (FB), the electric field is reversed, and the BPM converts the chemical potential energy of a pH gradient between an acid and a base to electrical energy via the recombination of protons and hydroxide ions into water at the BPM junction.^7^ The FB operation of BPMs has long received little attention compared to reverse bias, but this is significantly changing nowadays with the increasing interest in electrochemical systems and devices.^8,9^ In particular, for 1 M HCl and 1 M NaOH, the theoretical energy density is around 22 kWh for 1 m^3^ acid and 1 m^3^ base.^10^ This makes the FB operation relevant for increasing the discharge voltage of redox flow batteries, storing energy in a pH gradient (as is the case in the acid–base flow battery (ABFB)), or harvesting energy from waste acid and base streams using BPM reverse electrodialysis (RED).^7,11,12^

So far, most of the scientific work on BPM (R)­ED operation reports the performance of systems or membranes in pure acid and base streams that do not contain any contaminants.^7,13–15^ However, in real practical applications, the presence of salt in the acid and base cannot usually be excluded nor neglected.^10,16^ Furthermore, in systems where the acid and base are re-circulated, as is the case in e.g. flow batteries for energy storage such as the acid base flow battery (ABFB), background salt accumulation in the acid and base is inevitable due to the non-ideal BPM selectivity (< 100% permselectivity) that causes salt crossover.^17,18^ Pärnamäe and co-workers have shown that while the RB operation of the BPM is unaffected by the presence of background salt, the BPM FB voltage decreases with increasing background salt concentration in the acid and the base, which they attributed to the accumulation of Na^+^ and Cl^–^ ions at the BPM junction.^19^ The presence of these salt ions at the BPM junction lowers the availability of fixed charged groups to transport protons and hydroxide ions to the junction and reduces the available surface area for the water recombination reaction. Toh and coworkers observed similar results in mixtures of weak and strong acids and bases, and referred to the phenomenon as “ionic blockade”.^8^

The accumulation of Na^+^ and Cl^–^ ions at the BPM junction is due to the limited anion exchange layer (AEL) and cation exchange layer (CEL) selectivities for hydroxide ions over chloride ions and protons over sodium ions, respectively. That is not an issue in reverse bias since the ions are drawn away from the BPM junction. Fortunately, the smaller size and higher mobility of protons and hydroxide ions naturally favor higher H^+^/Na^+^ and OH^–^/Cl^–^ selectivities.^20^ However, in the case of the OH^–^/Cl^–^ couple, the differences between the two anion properties are small, substantiating that especially the BPM’s AEL suffers from the presence of background salt in the base compartment. Therefore, mitigating these ionic blockades requires preventing the transport of Cl^–^ ions to the junction in the first place by increasing the AEL’s OH^–^/Cl^–^ selectivity,^8,21^ which cannot be achieved with typical ion exchange polymers as these do not have a good selectivity between different monovalent ions.^22^

One property that distinguishes H^+^ and OH^–^ from other ions is their ability to hop from one water molecule to another via the so-called Grotthus mechanism,^23^ while other ions are only transported by diffusion, electromigration and/or advection.^22^ Therefore, a noteworthy way to increase the OH^–^/Cl^–^ selectivity is by leveraging the difference in the transport mechanism.

Poly­(benzimidazole) (PBI) polymers have widely been used in fuel cells, both as proton-conducting polymers in their acid-doped form^24^ and hydroxide-conducting materials in their alkaline-doped form,^25^ and have demonstrated promising properties. This is thanks to the amphoteric nature of the benzimidazole group that enables its protonation (pKa: 5.55) and deprotonation (pKa: 12.78).^26^ Most importantly, despite the negative charge of the deprotonated benzimidazole units in alkaline environments, OH^–^ ions rather than Na^+^ ions are the main ionic current carriers in such materials due to their higher mobility in the alkaline-doped free volumes, contrary to what is expected from a typical negatively charged cation exchange materials.^27^ Following this observation, we hypothesize that coating the BPM’s AEL with a thin PBI layer will increase the AEL OH^–^/Cl^–^ selectivity by allowing OH^–^ transport via hopping through the alkali-doped free volumes, but blocking Cl^–^ ions by Donnan exclusion.^28^

In this work, we first prove that the BPM forward bias performance is more sensitive to the background salt in the base than in the acid. Then, we investigate the effect of casting a thin and dense o-poly­(benzimidazole) layer on top of the AEL surface of a commercial BPM to increase its OH^–^/Cl^–^ selectivity. Finally, we report the electrochemical properties of the PBI-coated BPMs under FB in salt-contaminated acid and base to confirm our hypothesis; and we show that the coating indeed increases the peak power density for harvesting energy from a pH gradient in the presence of background salt, which is relevant for practical applications.

## Materials and Methods

### Materials

The commercial FBM BPM from Fumatech BWT GmbH (Germany) was used as bipolar membrane. For the membrane coating, absolute ethanol (> 99.5%, TechniSolv^®^) was purchased from VWR chemicals (The Netherlands), and o-poly­(benzimidazole) (PBI, Fumion AM) was purchased from Fumatech BWT GmbH (Germany). For the membrane characterization, sodium chloride (NaCl, Sanal^®^ P, pharmaceutical grade) was kindly supplied by Nouryon (The Netherlands). Sodium hydroxide (pellets, EMPLURA^®^) and hydrochloric acid (32%, EMPLURA^®^) were purchased from Merck (Germany). Sodium sulfate decahydrate (Na_2_SO_4_ • 10 H_2_O) was purchased from Thermo Fisher (Germany) and was used for the electrolyte. The commercial cation and anion exchange membranes used as auxiliary membranes in the six-compartment setup were Neosepta CMX-fg and AMX-fg membranes, respectively, from Astom (Japan). Demineralized water was obtained from an Elga Water Purification System from Veolia (The Netherlands). All chemicals were used as received.

### Membrane Modification

The commercial FBM BPM was modified by surface-coating the AEL with o-polybenzimidazole. A 3x3 cm^2^ wet FBM BPM piece was taped to a glass plate to prevent its curling using a 40 μm-thick tape on two edges (in the direction of the coating). Using a 0.15 mm casting knife, a PBI solution in ethanolic KOH was cast on top of the FBM membrane (see Table 1 for the solution composition). The excess PBI solution around the membrane sample was removed with tissue paper (the coating on the BPM surface was already dry), and the BPM was untaped and put in demineralized water for 15 min. Afterwards, the BPMs were stored in 0.5 M NaCl solution and their electrochemical performance was assessed after at least 24 h to prevent artifacts due to remaining KOH from the coating process. The BPMs are further referred to using the PBI and the KOH wt % of the solution used to coat the BPM.

**Table 1:** Composition of the PBI in Ethanolic KOH Solutions Investigated to Cast on the Fumatech FBM BPM’s AEL

wt % KOH in ethanol	wt % PBI	OH^–^:imidazole molar ratio
5 wt % KOH	1	14.34
	2	7.31
	3	4.85
10 wt % KOH	1	28.85
	2	14.52
	3	9.66
	4	7.26
20 wt % KOH	3	19.55
	4	14.47
	5	11.49

### Membrane Characterization

#### Electrochemical Characterization

The electrochemical performance of the BPMs was characterized using a six-compartment cell as depicted in the Supporting Information, Figure S1. The exposed BPM area was 0.785 cm^2^ (1 cm diameter). An SP-150e potentiostat from BioLogic was used to perform the measurements.

For the reverse and forward bias direct current measurements, a current density from 0 to 100 A/m^2^ in steps of 10 A/m^2^ was applied for two min per step (the time was adjusted in case the voltage was not stable within those 2 min). The measurements were always done after equilibrating the samples at open cell voltage (OCV) with the solutions for 10 min prior to the experiment (until the OCV stabilized). The current-density voltage curves were obtained by taking the average of two independently coated FBM BPM samples, and the error bars represent the standard deviation of the two samples. The uncoated commercial FBM BPM was tested between 0.5 M HCl and 0.5 M NaOH, with 0.1 M background NaCl in the acid and/or the base (so in the four cases: pure acid and base; only the acid or the base are salt-contaminated; and both acid and base are salt-contaminated). All the PBI-coated BPM samples were measured between 0.5 M HCl with 0.1 M NaCl and 0.5 M NaOH with 0.1 M NaCl (both the acid and the base were contaminated with background salt).

The power density and the voltaic efficiency were calculated from the obtained direct current data according to the following equations:

1 $$\mathrm{PD}=\frac{U\cdot I}{A_{\mathrm{BPM}}}$$

where PD is the power density (W/m^2^), U is the stabilized potential (V), I is the applied current (A) and A_BPM_ is the BPM area (m^2^), and:

2 $$\mathrm{VE}=\frac{U_{\mathrm{FB}}}{U_{\mathrm{RB}}}\cdot 100\%$$

where VE is the voltaic efficiency (in %), t is the time (s), U is the potential (V), and the subscripts FB and RB correspond to forward bias and reverse bias, respectively.

The electrochemical impedance spectroscopy (EIS) measurements were done at a current density of 80 A/m^2^ (applied current: 6.283 mA) with an amplitude of 10% (0.628 mA), in the same solutions as for the direct current measurements (reported in the previous paragraph). Before the EIS scan, the BPM was held at the desired current for two min after a 10 min stabilization of the BPM between the acid and base solution, to ensure steady state during the measurement. The EIS scan was performed from 100 kHz to 10 mHz with an average of six points per frequency and six measurements per decade. The EIS spectra were analyzed by using the Zfit and Zsim functions of the EC-lab software (version V 11.50).

The long-term FB operation of the BPMs was assessed by holding the BPM at a constant current density of 80 A/m^2^ for 3 days and monitoring the voltage over time.

#### Scanning Electron Microscopy

The BPM top surfaces and the cryogenically fractured BPM cross sections were observed with a JEOL JSM-IT100 scanning electron microscope (Jeol, The Netherlands). The samples were sputter-coated with gold for 70 s at 60 mA with a JEOL JFC-2300 HR sputter coater (Jeol, The Netherlands). The observation conditions were a 10.0 kV applied voltage, 10 mm working distance, and 60 nA probe current.

## Results and Discussion

### Influence of Background Salt on the Fumatech FBM BPM Forward Bias Performance

Published results in peer-reviewed journals provide evidence that BPMs are sensitive to background salts in the acid and base compartments due to the accumulation of salt ions at the BPM junction in forward bias.^10,16,19^ However, these studies considered only symmetrical cases in which the acid and the base were contaminated with the same NaCl concentration. In such conditions, one cannot identify which side of the BPM contributes most to the observed overpotential. Therefore, we report in Figure 1 the current-density voltage curves for a BPM under forward bias between pure 0.5 M HCl and NaOH, and in the presence of 0.1 M NaCl in the acid and/or base compartment(s).

**Figure 1:**
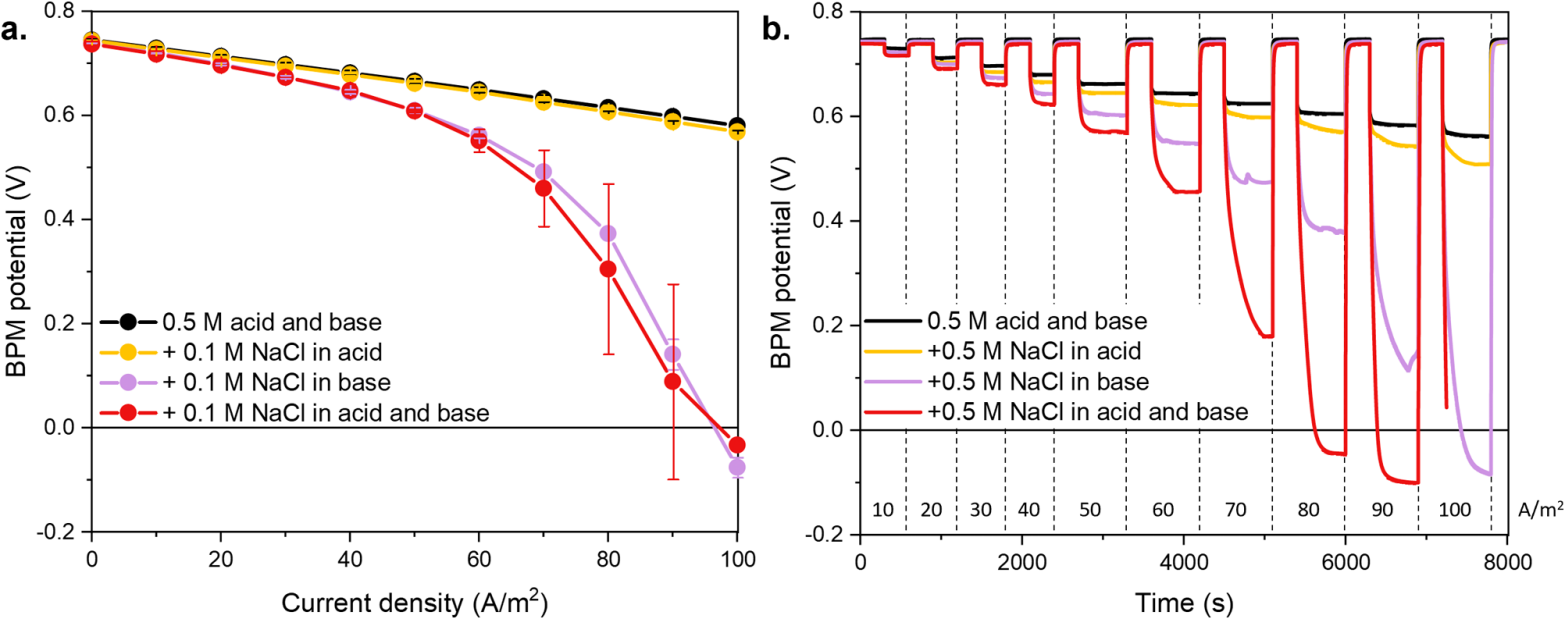
**a.** Current density-voltage curves of the commercial FBM BPM under forward bias in the presence of background salt in the acid or base. **b.** Chronopotentiometry of one of the FBM BPM samples in the different cases. The numbers correspond to the applied forward bias current density after 5 min of open cell voltage.

Figure 1a shows that while the case with pure 0.5 M acid and base follows Ohm’s law, other situations show clear deviations due to the presence of background salt, in line with the results of Pärnamäe et al.^19^ Indeed, as the current density increases, the overpotential for water recombination in salt-contaminated streams becomes larger than that in the case of pure acid and base. The increasing resistance with increasing current density indicates the non-ohmic nature of these losses.^29^ Figure 1b reports the chronopotentiogram of one of the BPM samples in the different cases, and supports the non-ohmic nature of the energy losses in the presence of background salts in the solution, as shown by the longer stabilization times. Combined with the unstable voltage, the longer stabilization times when operating the FBM BPM at high current density FB in salt-contaminated acid and base highlight the instability of these operating conditions, as reflected in the large error bars. These results indicate mass transport limitations within the BPM caused by the competing transport of salt ions toward the BPM junction and the operation at unsteady conditions.

Under FB in pure solutions, the H^+^ ions from the acid and the OH^–^ ions from the base recombine at the BPM junction. Their spontaneous recombination into a neutral water molecule (H_2_O) immediately clears the ionic pathways for new ions to be transported to the junction. However, if the solutions are salt-contaminated, part of the ionic current toward the BPM junction is carried by Na^+^ and Cl^–^ ions. These ions cannot neutralize and eventually accumulate at the junction where they hinder the transport of H^+^ and OH^–^ ions; both by reducing the amount of AEL and CEL available fixed charge groups due to charge condensation and by the additional Donnan exclusion created by the accumulation of similarly charged ions near the junction. In addition, the pairing between the AEL and CEL fixed charges and salt ions also leads to a reduction in the BPM potential, creating an additional overpotential penalty.^30^ Finally, the presence of these ions at the junction also physically reduces the volume available for water recombination to occur, and can block catalytical sites relevant for the water recombination reaction.^31^ The combination of these phenomena has been termed “ionic blockade” by Toh and coworkers, who reported similar results in the cases where the KOH compartment was contaminated with weaker bases such as acetate.^8^ Operating a BPM in the ionic blockade regime is undesirable, as it is an unstable operating window (as illustrated by the large error bars and the longer stabilization times) and creates large voltaic losses. Whether the Na^+^ and Cl^–^ ions recombine to form NaCl crystals at the junction is still uncertain. Blommaert et al. hypothesized the formation of potassium salts at a BPM junction in a CO_2_ electrolyzer,^32^ and Al-Dhubhani et al. suggested NaCl crystal formation in the BPM junction.^13^ This would mean that the NaCl concentration at the junction is locally higher than the solubility limit of NaCl in water (i.e. 360 g/L (6.16 M) at 25°C^33^). At these high salt concentrations, the Donnan exclusion of the AEL and CEL begins to fail, enabling Na^+^ and Cl^–^ crossover through the AEL and CEL, respectively.^34^ Moreover, the high osmotic pressure difference between the BPM junction and the acid and base solutions restricts water diffusion out of the junction and results in water accumulation in the junction, causing the BPM to delaminate before salt crystals start forming. Indeed, we observe delamination when we place the commercial FBM in forward bias in pure 0.5 M NaCl solutions (see supplementary information S2), but we do not detect defects when the BPM is between 0.5 M HCl and 0.5 M NaOH. Irrespective of salt crystal formation (which necessitates further studies beyond the scope of this work), the accumulation of these salt ions at the junction negatively impacts the performance and mechanical stability of the commercial FBM BPM.

Figure 1a and b also show that the FB overpotential in the presence of background salt is mainly influenced by the contamination of the base rather than the acid, which can partly be explained by the higher similarity between Cl^–^ and OH^–^ ions in terms of size and diffusion coefficient than between H^+^ and Na^+^ ions.^18,20,35^ While Na^+^ ions have a 1.3 times larger radius and their transport is 7 times slower than protons, chloride ions are only 1.1 times larger and 2.5 times slower than hydroxide ions.^18^ It naturally follows that the AEL selectivity for OH^–^ over Cl^–^ (OH^–^/Cl^–^) is lower than the CEL selectivity for H^+^ over Na^+^ (H^+^/Na^+^).^22^ In addition, H^+^ benefit from their smaller size and their twice as fast diffusivity compared to OH^–^, making H^+^ less sensitive to the presence of ionic blockades.^20,35^ Whether the better CEL H^+^/Na^+^ selectivity or the higher mobility of protons leads to these results necessitates further studies relying on lab-made BPMs beyond the scope of this study. Nonetheless, the results undeniably point to the higher sensitivity of the AEL side to the ionic blockade hindering the transport of OH^–^ ions. Therefore, accumulation of Cl^–^ ions near the BPM junction hinders the transport of OH^–^ ions, which leads to a limiting current density around 70 mA/cm^2^ as the electric current becomes limited by diffusion limitations, and increasing the potential does not lead to a large current density increase.

To better understand the cause for the observed increased overpotential, electrochemical impedance spectroscopy (EIS) results measured above the limiting current density are reported in Figure 2. The equivalent circuit chosen to fit the Nyquist plot is displayed in Figure 2a, and consists of a resistor (R) for the ionic resistance, one RC (resistor–capacitor circuit) for the water association at the BPM junction, and one RC for the transport in the double layers.^36^

**Figure 2:**
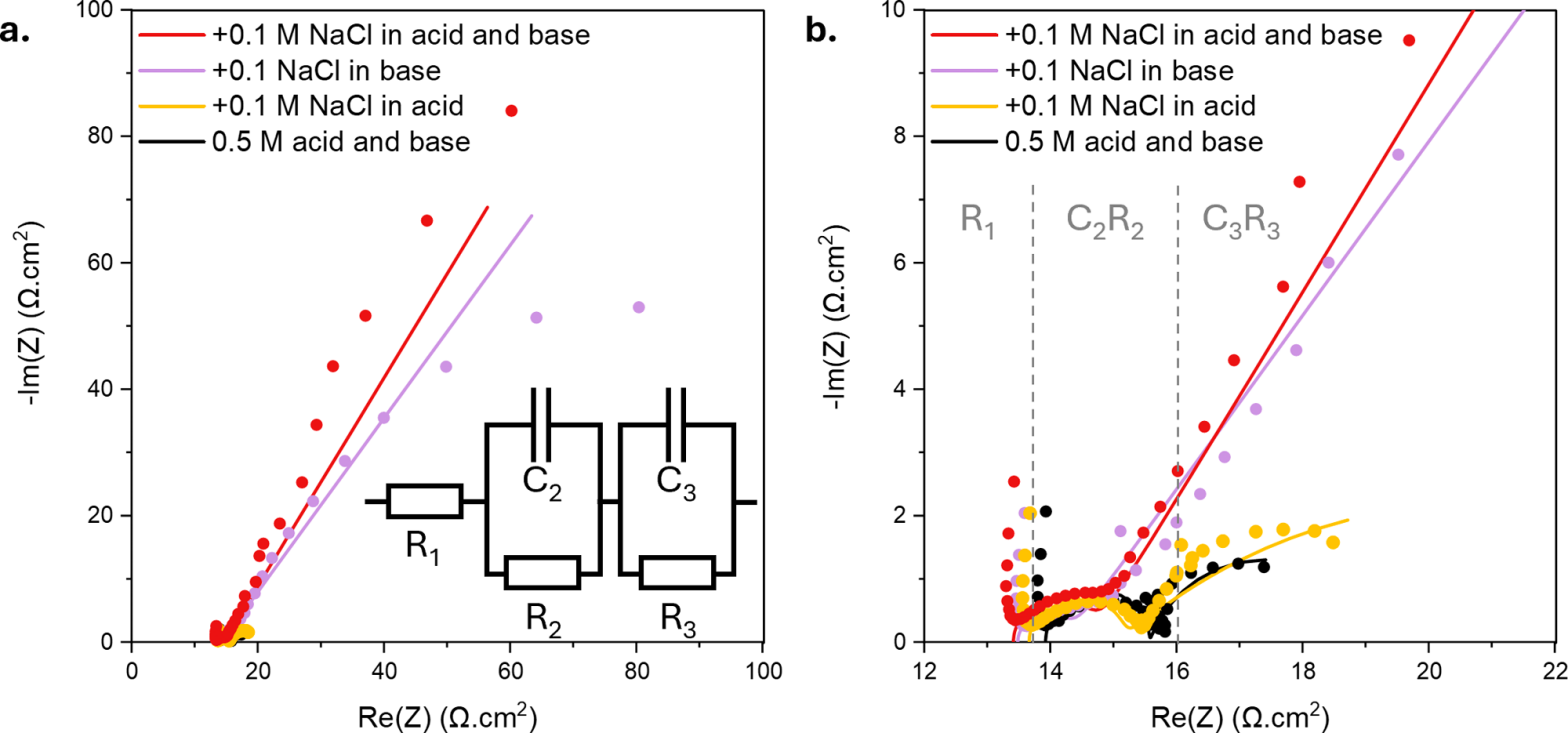
EIS spectra of the commercial FBM BPM in acid and base and in the presence of background salt in acid and/or base. **a.** Full Nyquist plot and equivalent circuit used to analyze the data. **b.** Zoom into the high-frequency region. The dots correspond to the measured values, and the lines correspond to the simulated equivalent circuit.

The Nyquist plot shapes in Figure 2a and b support the idea that the presence of background salt in the base induces mass transport limitations, as shown by the much larger second arc at low frequencies when the base is salt-contaminated compared to when a clean 0.5 M NaOH solution is used. Quantitative analysis is performed by fitting the Nyquist plots to an equivalent circuit. Choosing an equivalent circuit is a critical step in the Nyquist plot interpretation, as several circuits can be used to fit the same data. In our case, we chose a Voigt circuit with two RCs in series due to the presence of two semi-circles. This circuit is similar to what is chosen in literature for modeling the water association reaction in BPMs,^36^ and more generally, electrochemical reactions.^37^ Interestingly, the chosen Voigt circuit fit is poorer in the cases where the base is contaminated with 0.1 M NaCl. Looking at the Bode plot of the case in which both acid and base contain background salt (supplementary information S3), a reasonable consideration is replacing the RC circuit related to mass transport (R_3_C_3_) with a Warburg element, which is better suited for the description of semi-infinite diffusion.^37,38^ This is justified by the rather constant phase of -45° at low frequency, as well as the -1/2 slope for the impedance logarithm (supplementary information S3). However, a similar data interpretation of all the different cases enables a fairer comparison, and therefore, all the Nyquist plots are fitted with the equivalent circuit displayed in Figure 2a The thus extracted equivalent circuit values are reported in Table 2.

**Table 2:** Extracted Values of the Equivalent Circuit Fit Shown in Figure 2

	Pure acid and base	+0.1 M NaCl in acid	+0.1 M NaCl in base	+0.1 M NaCl in acid and base
R_1_ (Ω·cm^2^)	13.92 ± 0.13	13.72 ± 0.10	14.18 ± 0.16	14.17 ± 0.13
C_2_ (10^–4^ F)	4.65 ± 0.77	4.76 ± 0.66	249 ± 0.33	414 ± 48
R_2_ (Ω·cm^2^)	1.65 ± 0.12	1.89 ± 0.12	7.21 ± 0.63	10.59 ± 1.10
C_3_ (F)	1.90 ± 0.10	1.01 ± 0.00	0.09 ± 0.00	0.13 ± 0.01
R_3_ (Ω·cm^2^)	3.62 ± 0.35	3.54 ± 0.30	97.87 ± 5.84	148.64 ± 18.41

Table 2 shows that the ohmic resistance R_1_ (mainly governed by the bulk conductivity of the CEL and AEL and the solutions) is very minimally affected by the ionic blockade which only hinders the transport of reactants (OH^–^ and H^+^) at the junction. On the other hand, the C_2_ capacitance largely increases when background salt is present in the base. A larger capacitance indicates a larger ability to store charges and a narrower ion depletion region. Typically, during water recombination, the capacitance of the BPM junction should be as low as possible, since protons and hydroxide ions recombine at the junction, creating a neutral region. However, when Cl^–^ ions accumulate at the junction, they cause a larger capacitance and a narrower electrically neutral region.

Along with the C_2_ increase, the R_2_ and R_3_ resistances increase when a background salt is present, especially in the base. These resistances are related to the water association resistance (R_2_) and the mass transport of reactants (OH^–^ and H^+^) towards the junction (R_3_). The Cl^–^ ions accumulation at the junction reduces the availability of fixed charges and electrostatically repels the similarly-charged OH^–^ ions, substantially increasing the mass transport resistance of OH^–^ to the junction (R_3_). Consequently, the water association resistance (R_2_) increases as well since the OH^–^ concentration at the junction decreases, which decreases the water association rate following Le Chatelier’s principle.^31^ Therefore, the Cl^–^ ionic blockade not only increases the OH^–^ mass transfer resistance, but also the recombination rate as it decreases the availability of reactants (OH^–^ ions) at the junction. Typically, mass transport limitations occurring at a surface in contact with a liquid electrolyte can be mitigated by increasing the stirring to decrease the diffusion boundary layer thickness.^39^ However, in a BPM, the transport limitations occur at the junction of two solid layers (AEL and CEL) and are challenging to mitigate by modifying the operating conditions, highlighting the need for better-designed BPMs.

### Coating of the Commercial Bipolar Membrane’s AEL with o-Poly­(benzimidazole) Dissolved in Ethanolic KOH: Coating Morphology

To address the root cause of the problem, the AEL (which is predominantly responsible for the ionic blockade) is coated with a thin OH^–^/Cl^–^ selective layer to prevent the accumulation of Cl^–^ ions at the BPM junction.

As the coating is made with a volatile solvent (ethanol) and a high concentration of salt (KOH) on a wet surface (the BPM’s AEL), coating a wet FBM BPM with PBI solutions in ethanolic KOH is a complex process that involves: ethanol and KOH diffusion into the AEL, water diffusion from the AEL into the cast polymer film, ethanol evaporation from the top surface of the film, and the potentially resulting formation of KOH crystals that can act as pore-forming agents in the top part of the film. These phenomena are summarized in the schematic reported in supplementary information S4.

To determine the best coating conditions, the morphology of the coatings obtained with the different solutions (reported in Table 1) is observed by using SEM. The results are reported in Figure 3.

**Figure 3:**
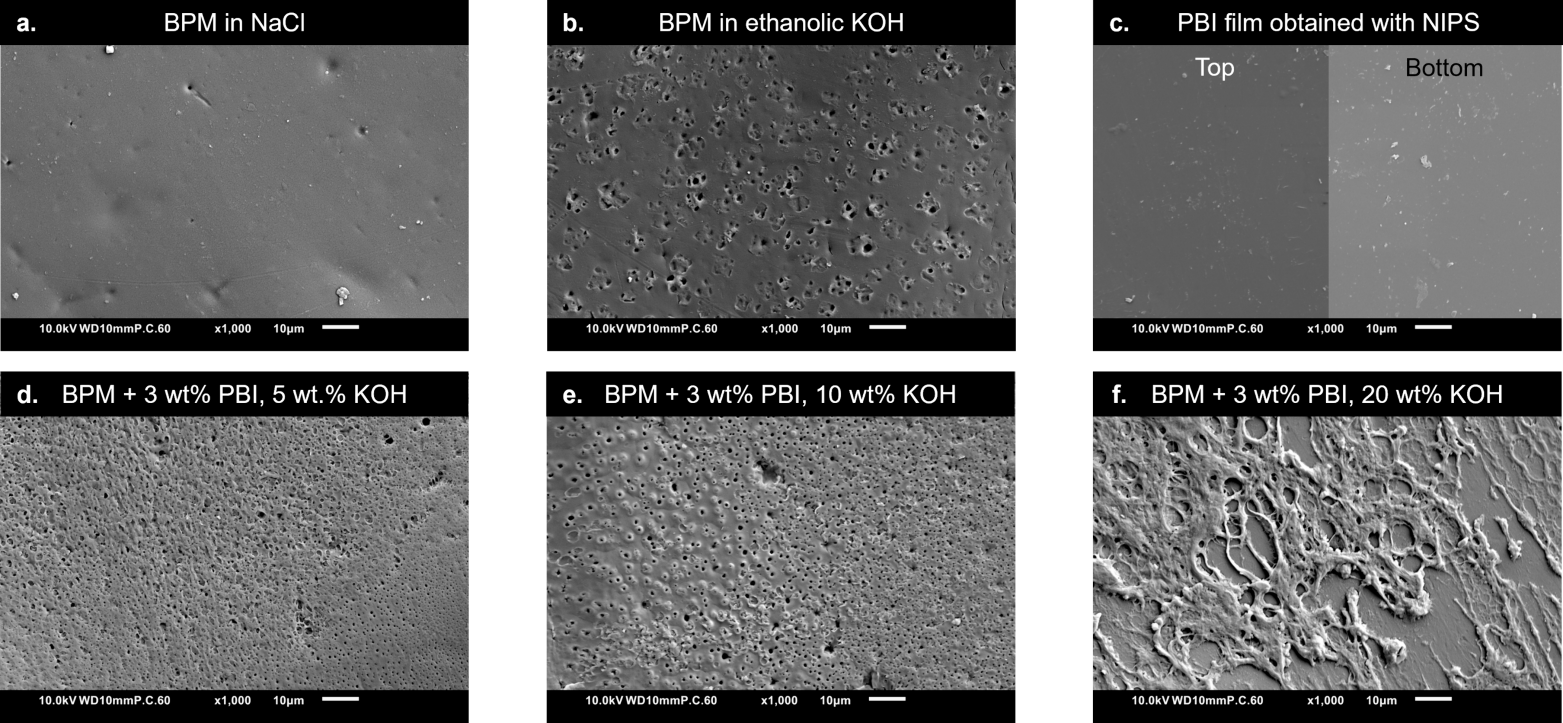
AEL surface view of the following samples: **a.** uncoated FBM BPM, **b.** uncoated FBM left for 15 min in 10 wt % KOH in ethanol, **c.** self-standing PBI film cast from 3 wt % PBI in 10 wt % KOH in ethanol similarly to the BPM coatings, **d.** FBM coated with 3 wt % PBI in 5 wt % KOH in ethanol, **e.** FBM coated with 3 wt % PBI in 10 wt % KOH in ethanol, **f.** FBM coated with 3 wt % PBI in 20 wt % KOH in ethanol.

As expected from an ion exchange layer, the AEL surface of an uncoated BPM (Figure 3a) is relatively smooth and dense (non-porous). The BPM surface kept for 15 min in a 10 wt % KOH solution in ethanol displays physical degradation of the AEL surface (Figure 3b), as ethanol induces FAA-3 swelling,^40^ the polymer used to manufacture the BPM’s AEL.^41^ FT-IR spectra of the FBM BPM and FAA-3 further confirm the BPM’s AEL composition (see supplementary information S5). In addition, the high KOH concentration creates a corrosive environment that degrades the AEL.^42^ The resulting surface degradation is not detrimental to the BPM performance, as shown in the next section. Furthermore, since the coating process lasts less than two min (after which the BPM is immersed in water), it is expected that the coated BPMs are less degraded than the sample in Figure 3b, as can be seen in the bare parts of the BPM coated with 3 wt % PBI and 20 wt % KOH (Figure 3f).

The self-standing PBI film cast in a similar way as the coating on the BPM (0.15 mm casting knife on top of 40 μm-thick tape, followed by immersion of the polymer film in demineralized water) displays a dense top and bottom surface (Figure 3c). The dense structure observed on both sides of the PBI film is exceptional for non-solvent induced phase separation (NIPS)-made membranes, as these membranes typically have asymmetric morphologies with a thin skin layer at the top with a gradual transition towards an increasingly porous structure.^43^ The fully dense morphology observed here is attributed to the high volatility of ethanol, resulting in high evaporation rates, inducing fast concentration and densification of the polymer film. The resulting increase in chain entanglement and solution viscosity leads to a dense morphology free of macrovoids.^44^ Therefore, the cast film is rather made by solvent-evaporation induced phase separation (SIPS) than NIPS, or a combination of first SIPS then NIPS, where the remaining solvent is washed away in the non-solvent water bath.^45^ Associated with that is the effect of the film thickness: the polymer solution film cast here is rather thin, whereas cast membranes are in general much thicker. In such thicker films, the top and bottom of the film generally experience different environments, typically resulting in a denser structure at the top and a more porous structure at the bottom of the film.^46^ This is also confirmed by the porous structure formed when casting the same 3 wt % PBI solution in 10 wt % KOH in ethanol but with a significantly thicker (0.5 mm instead of 0.15 mm) casting knife (see the supplementary information S6 for the corresponding optical and SEM images). To coat the BPM’s AEL, a dense layer displaying ion/ion selectivity is required, and the casting conditions are chosen such that a dense layer is indeed formed, as shown in Figure 3c

Surprisingly, Figure 3d and e show that the top surface morphologies of the coatings obtained at 3 wt % PBI are porous when using 5 and 10 wt % KOH in ethanol, contradicting Figure 3c. This observation is attributed to the high volatility of ethanol, which leads to fast solvent evaporation from the air-facing film surface. As ethanol evaporates, KOH starts crystallizing and then acts as a pore-forming agent. Upon immersion in water, the KOH crystals dissolve and create the observed porous structure. The KOH crystals observed at the surface of a free-standing PBI film produced by the evaporation of ethanol at ambient conditions (instead of immersing the film in water) further support this theory (supplementary information S6). Finally, the coatings with 20 wt % ethanolic KOH (Figure 3f) do not homogeneously cover the AEL surface due to the high solution viscosity with increasing KOH content.^47^

Provided a uniform polymer coating at 5 and 10 wt % KOH in ethanol, PBI coating on a wet BPM membrane leads to a dense film. While the AEL-PBI interface undergoes densification due to ethanol evaporation, the fast ethanol evaporation at the coating-air interface causes KOH crystallization, which creates a porous upper structure. These pores do not span the whole coating thickness, and the bottom part of the coating is dense. To provide additional proof that the PBI-AEL interface consists of a dense PBI film (as expected from the top and bottom part of the self-standing PBI film obtained in Figure 3c), the cross-section of the FBMs coated with 3 or 4 wt % PBI in 10 wt % KOH are reported in Figure 4.

**Figure 4:**
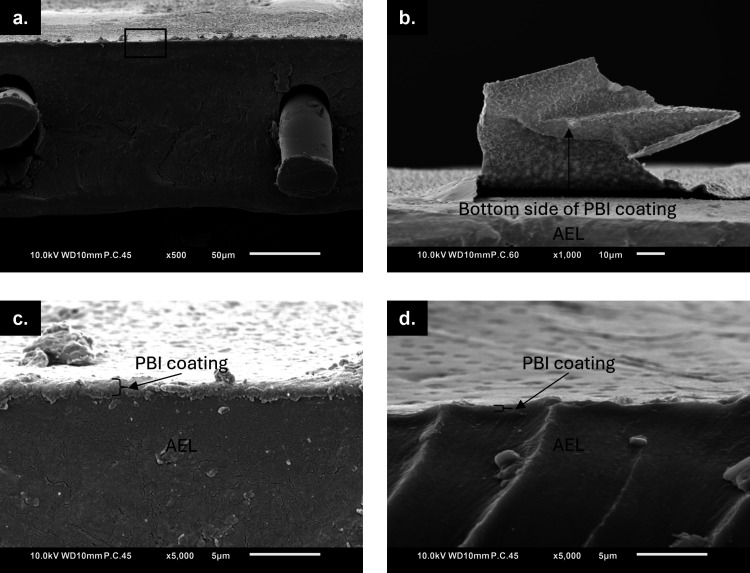
SEM cross sections of the BPM coated with **a.** 4 wt % PBI in 10 wt % KOH at 500× magnification, **b.** 4 wt % PBI in 10 wt % KOH at 1000× magnification on a detached coating piece, **c.** 4 wt % PBI in 10 wt % KOH, 5000× magnification on the square in Figure 4a, **d.** 3 wt % PBI in 10 wt % KOH, 5000× magnification.

First of all, the intact cross-section in Figure 4a does not show visible degradation of the BPM’s AEL thanks to the short contact times with ethanolic KOH. Coincidentally, part of the coating cast from a 4 wt % PBI solution in 10 wt % ethanolic KOH became loose upon cryogenically breaking the sample, exposing the bottom of the coating (Figure 4d), and revealing a dense but rough surface. Zooming further onto the coating, Figure 4c reveals that even with the highest PBI concentration, the obtained coating is very thin (in the order of one micrometer) due to the very low PBI concentration (4 wt %), and the thin casting thickness (0.19 mm in total including the tape). Furthermore, Figure 4c displays a dense coating cross-section, confirming that the observed “pores” at the coating-air interface of the coated BPMs are only superficial and do not span the full coating thickness. Finally, Figure 4d shows that the thickest coating (3 wt % PBI) obtained for the 5 wt % ethanolic KOH solution is even thinner than the one displayed in Figure 4c due to the lower polymer concentration and the resulting lower solution viscosity.

### Electrochemical Performance of the PBI-Coated BPMs in Salt-Contaminated Acid and Base

Finally, the electrochemical performance of the PBI-coated BPMs is evaluated to analyze the effect of the coating on the BPM performance and resilience to the presence of background salt in the acid and base in FB. Figure 5 displays the current density-voltage curves of the differently coated BPMs.

**Figure 5:**
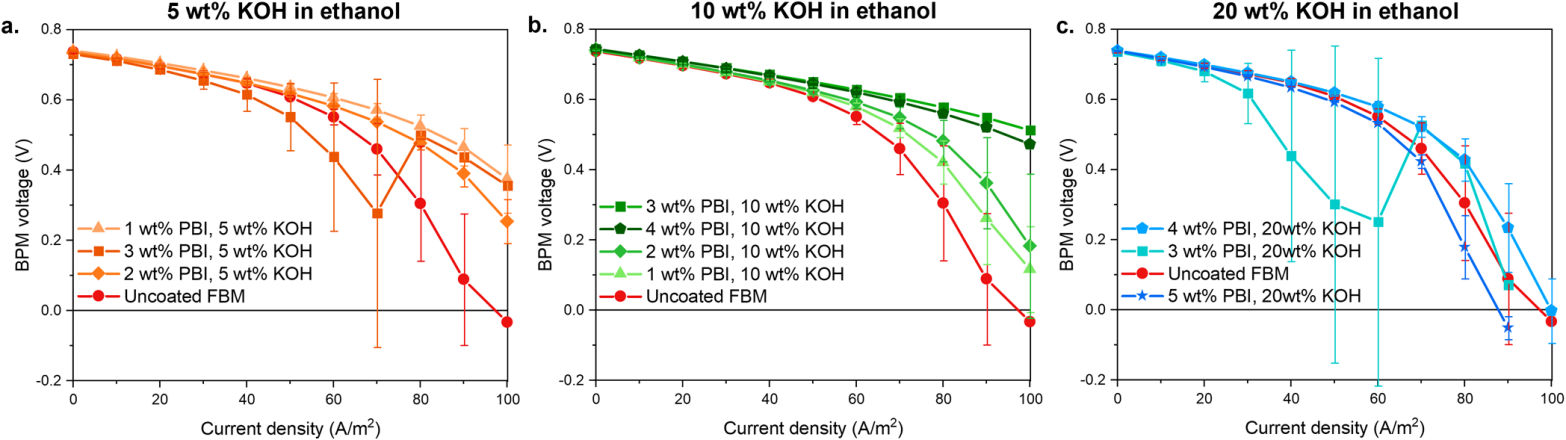
Current density-voltage curve of the PBI-coated FBM BPM with **a.** 5 wt % KOH solution in ethanol, **b.** 10 wt % KOH solution in ethanol, and **c.** 20 wt % KOH solution in ethanol. To guide the reader, the legends are ordered in the same order as the data points appear from top to bottom.

When 5 wt % KOH in ethanol is used as coating solvent, only a minimal improvement in performance for the coated BPM compared to the uncoated BPM is observed (Figure 5a), due to the low viscosity of the 5 wt % KOH solutions that leads to thinner coatings (Figure 4d), and a faster ethanol evaporation rate that induces a larger surface porosity (Figure 3d). On the other hand, Figure 5b shows that when using 10 wt % KOH in ethanol as a solvent, increasing the PBI concentration improves the FB BPM performance. Increasing the PBI concentration not only increases the total coating thickness,^48^ but also the solution viscosity which leads to a thicker portion of dense OH^–^/Cl^–^ selective layer.^49^ As the PBI wt % increases from 3 to 4 wt %, no further enhancement of the BPM performance is noticed, i.e. increasing the coating thickness does not further increase the selectivity. In fact, the thicker coating increases the Ohmic resistance and causes a higher overpotential, lowering the BPM voltage. Finally, Figure 5c shows that coating the AEL with PBI dissolved in 20 wt % KOH in ethanol does not lead to performance enhancement as no homogeneous coating can be formed, as shown in the SEM image in Figure 3f.

The enhanced BPM FB performance when the BPM’s AEL is coated with PBI in 10 wt % KOH in ethanol solution is attributed to a higher OH^–^/Cl^–^ selectivity that originates from several factors. First, the strong interactions between PBI and FAA-3 densify the AEL-PBI boundary and therefore improve the size-exclusion of Cl^–^ over OH^–^. Such strong interactions between these two polymers are reported in literature that describes precautions to be taken to prevent the gelation of FAA-3 and PBI mixtures.^50^ The strong attractive forces are due to π-π interactions between the aromatic backbones of both polymers (PBI and FAA-3), and the electrostatic attraction between the negatively charged deprotonated imidazole groups and the positively charged quaternary ammonium groups that lead to ionic crosslinking. This hypothesis is supported by the reported preparation of acid–base blended membranes, where the acid–base pair formed between PBI and a proton exchange resin improves proton selectivity over, for example, vanadium ions.^51^ The ionic crosslinking between FAA-3 and PBI contains the water uptake of the AEL surface and therefore contributes to an enhanced OH^–^/Cl^–^ selectivity.^52^

In addition, the Donnan exclusion of Cl^–^ by the negatively charged deprotonated benzimidazole units also contributes to the enhanced performance of the BPM.^53^ A 0.5 M NaOH solution has a pH of 13.70, while the benzimidazole units have a pKa of 12.78, suggesting that the PBI coating is negatively charged at the operating pH.^26^ Despite these negative charges, previous literature has shown that the transport numbers of Na^+^ and OH^–^ ions in a NaOH-doped PBI membrane used in alkaline direct oxidation fuel cells are 0.28 and 0.72, respectively.^27^ This is due to the higher mobility of OH^–^ ions compared to Na^+^ ions, and their ability to hop between the alkaline-doped free-volume elements of PBI. Hence, as the negative charge of the deprotonated PBI units repels Cl^–^ ions by Donnan exclusion, the OH^–^ ions can still permeate through the PBI coating by hopping between the alkaline-doped free volumes.^53^

Therefore, when the PBI coating is successfully applied on top of the AEL’s surface as is the case for 3 and 4 wt % PBI solutions in 10 wt % KOH in ethanol, the Cl^–^ ionic blockade at the BPM junction is suppressed since the coating enhances the OH^–^/Cl^–^ selectivity of the AEL. As a result, the BPM operation at high current densities FB in salt-contaminated acid and base is more stable (as illustrated by the smaller error bars) and presents less energy loss.

To further understand the improved performance achieved for the BPM coated with a thin PBI film, EIS of the coated BPMs was performed, and the obtained Nyquist plots were analyzed with a similar circuit as described in Figure 2. Figure 6 reports the extracted fitted values, and the Nyquist plots are reported in the supplementary information S7.

**Figure 6:**
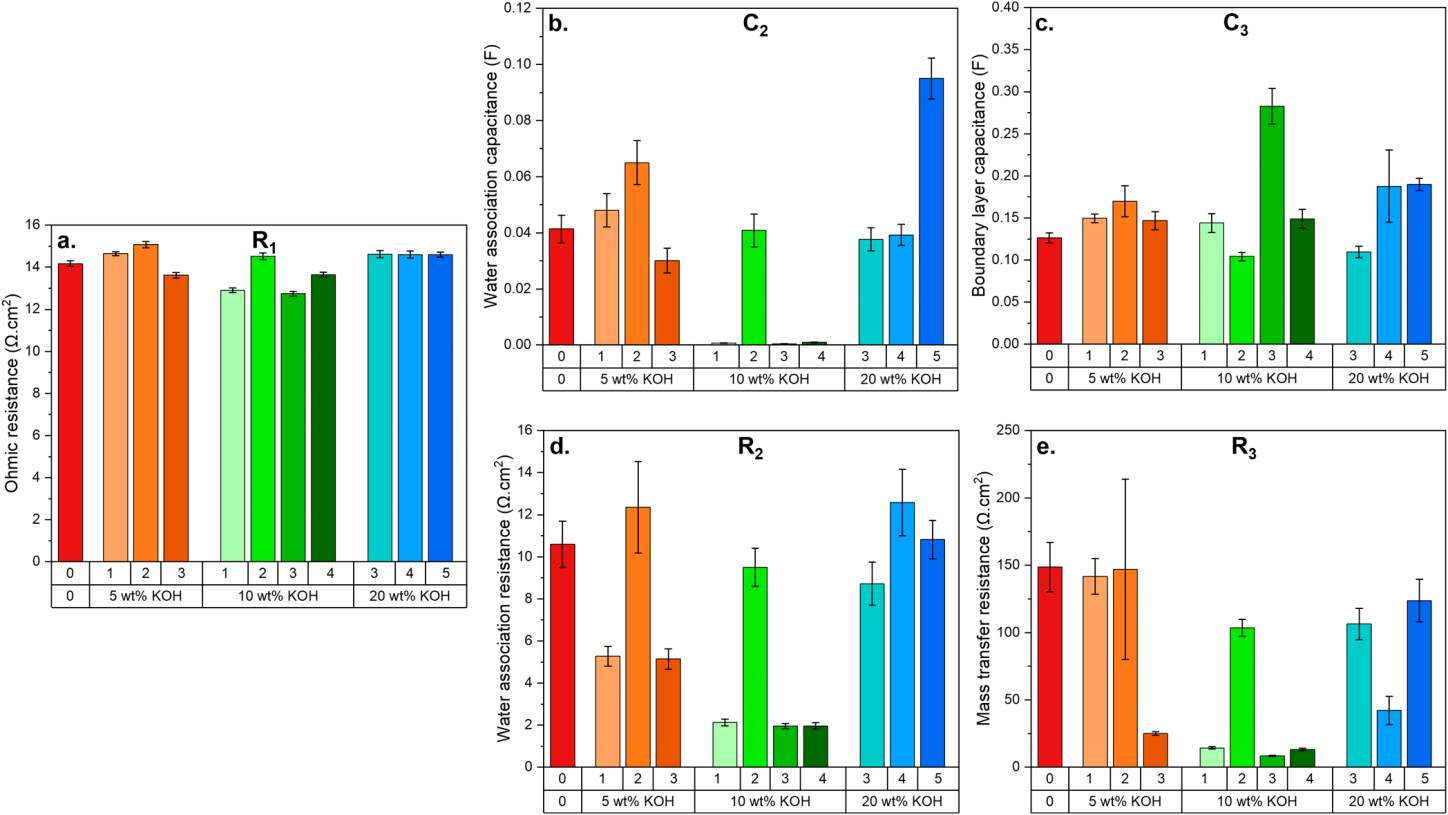
Values extracted from the Nyquist plot for the different coated FBM membranes. **a.** R_1_ (Ohmic resistance), **b.** C_2_ (water association reaction capacitance), **c.** C_3_ (boundary layer capacitance), **d.** R_2_ (water association resistance), **e.** R_3_ (mass transfer resistance). Measurements were performed at 80 ± 8 A/m^2^. The numbers 0, 1, 2, 3, and 4 used in the labels of the *x*-axes refer to the PBI wt % used in the coating solutions.

Figure 6a shows minimal changes in the Ohmic resistance R_1_, as the very thin selective layer does not add substantial ohmic resistance. Interestingly, Figure 6b and d show that the water association capacitance and resistance are greatly reduced by the introduction of a successful coating (i.e., 3 and 4 wt % PBI coating in 10 wt % KOH in ethanol). The 1 wt % PBI coating in 10 wt % KOH also has a low C_2_ and R_2_, but these samples display a large variability (Figure 7) showing that these coating conditions are therefore not the most optimal. The lower C_2_ and R_2_ values for the successfully coated samples are due to the higher OH^–^ concentration in the junction, which enhances the water association reaction rate and leads to an electrically neutral junction as OH^–^ ions are neutralized by protons upon water formation. Figure 6c shows that the effect of the coating on the capacitance next to the junction is rather negligible in most cases, which contradicts the findings in Table 2. However, the differences in C_3_ are not as striking as the other fitted parameters (C_2_, R_2_, and R_3_), and they could also be artifacts from the fitting and the assumption that the capacitors behave ideally. Finally, since the ionic blockade is prevented by the Donnan exclusion of Cl^–^ by the negatively charged PBI coating, the mass transfer resistance R_3_ of OH^–^ towards the junction is drastically lowered (Figure 6e).

**Figure 7:**
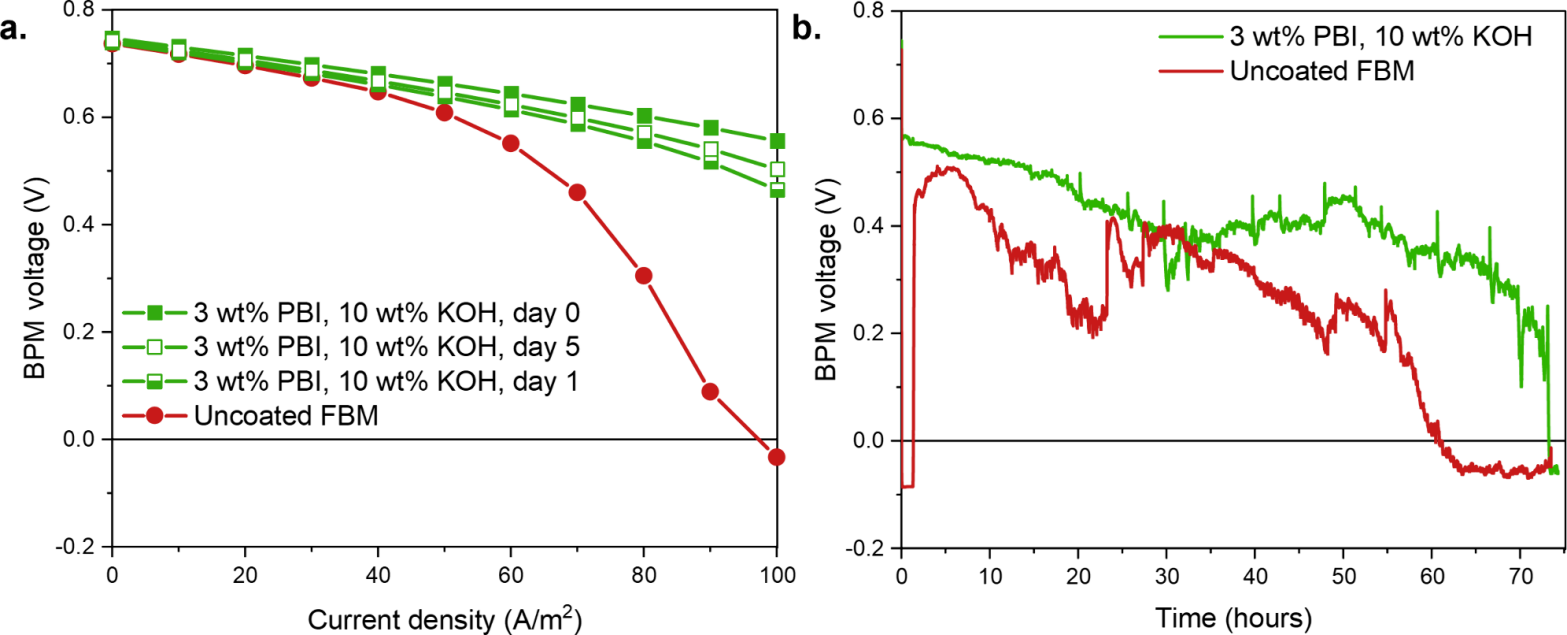
Long-term stability of the FBM coated with 3 wt % PBI in 10 wt % KOH in ethanol measured as **a.** the reproducibility of the measurements over time and **b.** the voltage stability over 3 days at a constant forward bias of 80 A/m^2^.

In short, a PBI coating made with 3 or 4 wt % PBI in 10 wt % KOH in ethanol applied on the AEL surface successfully mitigates the formation of a Cl^–^ ionic blockade when the BPM is in forward bias in salt-contaminated acid and base. The Donnan exclusion introduced by the coating layer prevents Cl^–^ accumulation near the BPM junction, which enables the unhindered transport of OH^–^ to the junction. The resulting higher OH^–^ concentration at the junction facilitates the water recombination reaction, leading to higher forward bias voltages.

### Long-Term Stability of the PBI-Coated BPMs

Since the coatings are made from a solution with a high KOH concentration, it is necessary to ensure that the observed behaviors are indeed attributed to the presence of the OH^–^-selective PBI coating and are not temporary artifacts from the AEL doping with OH^–^ ions. To do so, the reproducibility of the measurements over time and the voltage stability over 3 days at a constant forward bias of 80 A/m^2^ was assessed (Figure 7).

First of all, the reproducibility of a sample over time was assessed by measuring the same sample performance on different days (Figure 7a), where the sample was stored in 0.5 M NaCl between the measurements. Between the same day as the coating (day 0) and the day after (day 1), there is a noticeable decrease in the BPM voltage in forward bias, suggesting an effect of the AEL OH^–^-doping upon coating. In the later days (days 1 and 5), the forward bias performance is very similar, indicating that the effect of KOH doping no longer plays a role and does not influence the obtained results. That also confirms that storing the samples in 0.5 M NaCl solution for at least 24 h after coating is a good approach to ensure that the obtained electrochemical results are not artifacts from the remaining KOH in the AEL.

In addition, the longer-term stability of the BPMs was assessed by subjecting an uncoated and a 3 wt % PBI in 10 wt % ethanolic KOH-coated FBM BPM to an 80 A/m^2^ FB for 3 days (Figure 7b). The uncoated BPM potential is negative during the first minutes, in concordance with Figure 1. Surprisingly, after 82 min, the potential jumps to a positive value with a maximum of 0.51 V at 4 h, contradicting the findings in Figure 1, and suggesting that the ionic blockade “resolves” itself after a few hours, most probably thanks to water accumulation. Indeed, the water produced in FB accumulates over time and is further retained by the high osmotic pressure at the BPM junction caused by the accumulated Cl^–^ ions. The accumulation of water in turn leads to swelling of the BPM junction, enabling better transport of OH^–^ toward the junction and therefore better FB performance. However, despite temporarily improving the voltage, water accumulation at the BPM junction leads to a highly unstable system with a steady decrease in potential. The voltage eventually drops to negative values after 60 h of operation, demonstrating that the development of BPMs with better water management in FB is necessary to achieve long-term operation stability. This long-term instability is further supported by the highly fluctuating voltage observed as well for the coated sample after a day of operation, and by Xia et al., who observed a self-accelerating voltage drop at FB current densities above 40 A/m^2^ and attributed it to the accumulation of water at the BPM junction.^54^

In the case of the coated BPM, the obtained chronopotentiogram shows that a stable, yet steadily decreasing voltage can be obtained for a day-long operation. Afterwards, however, the potential becomes highly unstable as reported for the uncoated FBM BPM and eventually drops below zero after 73 h of operation. Re-starting the measurement with the same PBI-coated sample and with fresh solutions restores the operation and leads to a stable potential value that is similar to what is observed in the first hours reported in Figure 7b. These results show that the PBI coating improves the BPM performance for a day of operation at 80 A/m^2^ FB in salt-contaminated acid and base. However, after longer operating times, the inherent BPM instability which we attribute to water accumulation at the junction becomes problematic and highlights the need to develop better BPMs. This observed long term instability necessitates further studies on the interplay between electrochemical and transport phenomena to guide the development of BPM junctions with better water management.

### Implications for Bipolar Membrane Reverse Electrodialysis Processes

A reduced sensitivity to the presence of background salt is highly interesting for processes in which the BPM is operated in forward bias, such as energy harvesting from a salt-contaminated acid and base via bipolar membrane reverse electrodialysis. Figure 8 displays the discharge power density for the differently coated BPMs as extracted from the current density-voltage curves and the voltaic efficiency profiles of an uncoated and a PBI-coated FBM BPM.

**Figure 8:**
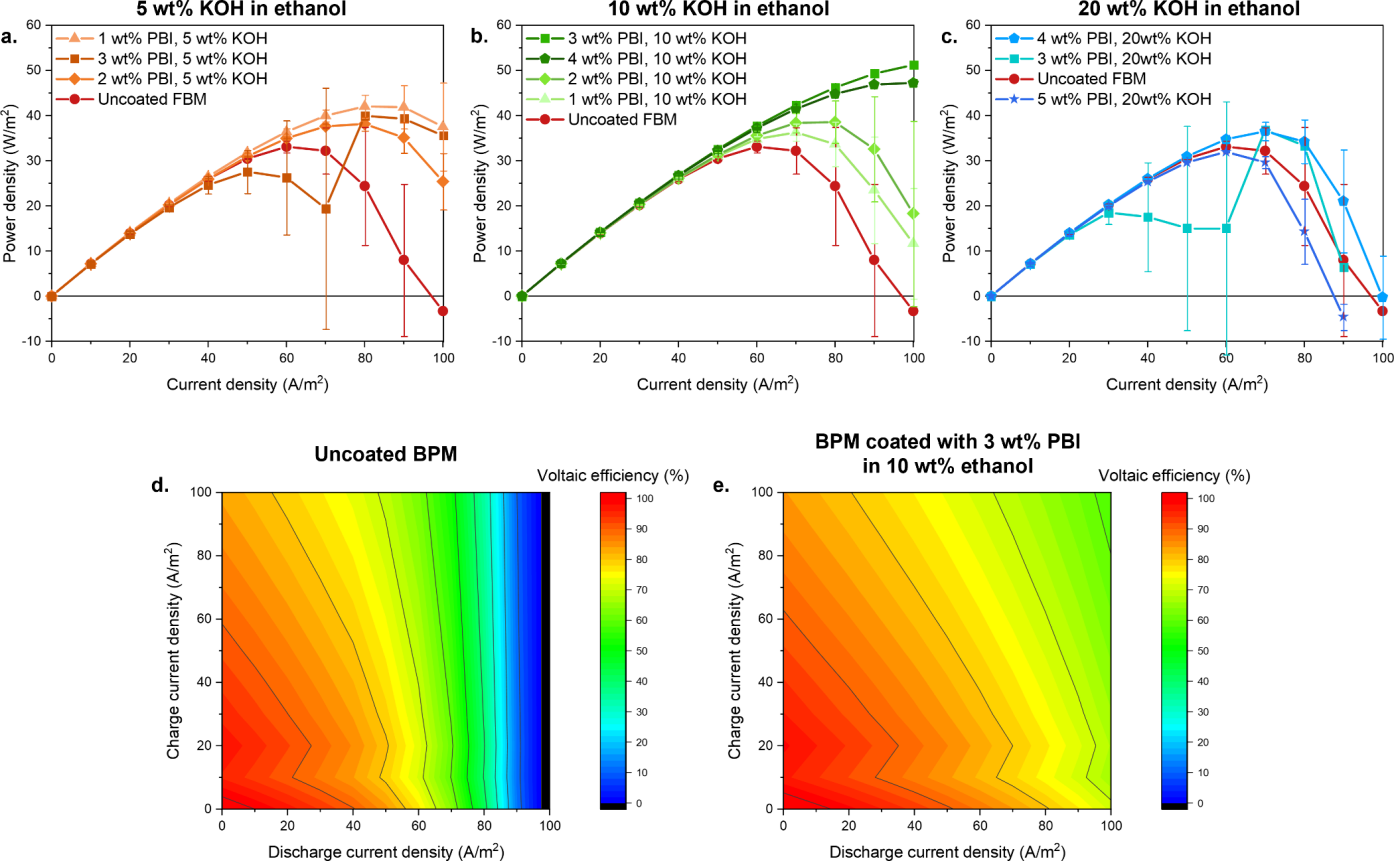
**a., b.** and **c.** Power density harvested vs. current density from the pH gradient between solutions of 0.5 M HCl and 0.5 M NaOH, both contaminated with 0.1 M NaCl for the differently coated BPMs. **d.** Voltaic efficiency profile of an uncoated FBM BPM between 0.5 M HCl and 0.5 M NaOH with 0.1 M NaCl in both compartments. **e.** Voltaic efficiency profile of an FBM coated with 3 wt % PBI in 10 wt % KOH between 0.5 M HCl and 0.5 M NaOH with 0.1 NaCl in both compartments.

The data in Figure 8a, b, and c show the expected trends based on Figure 5. While the uncoated BPM has a maximum power density of 32 W/m^2^ at 60 A/m^2^, a successful PBI coating increases both the maximum power density and the corresponding current density. The highest power density exceeds 51 W/m^2^ and is achieved with the 3 wt % PBI in 10 wt % KOH at 100 A/m^2^. This very significant increase is due to the reduced BPM sensitivity to background salts: the OH^–^/Cl^–^ selective PBI coating unlocks higher power densities for energy retrieval from salt-contaminated acid and base.

An improved FB performance in the presence of background salts is also relevant for applications such as the acid–base flow battery, in which excess renewable energy is stored in a pH gradient via BPM electrodialysis, and is later retrieved by BPM reverse electrodialysis.^12^ While these systems usually operate with pure acid and base streams, the inevitable crossover of ions through the membranes eventually leads to the accumulation of background salt in the acid and base solutions.^18^ This is detrimental to the long-term battery operation as the discharge power density becomes lower over time (as shown in Figures 8a, b, and c.), and the voltaic efficiency decreases.

Based on the measured BPM potential in reverse and forward bias in 0.5 M HCl and NaOH (both contaminated with 0.1 M NaCl), the BPM voltaic efficiency is reported in Figure 8d and e for the uncoated FBM and the FBM coated with 3 wt % PBI in 10 wt % KOH in ethanol.

Figure 8d and e clearly show that upon application of a PBI coating, the voltaic efficiency of the system increases. As expected, Figure 8d and e also shows an increasing voltaic efficiency with a decreasing (dis)­charge current density due to the higher overpotential losses at higher current densities.^29^ In the case of an uncoated BPM, it is preferential to avoid discharging the battery at current densities higher than 70 A/m^2^ to prevent a voltaic efficiency below 50%, while the BPM coated with 3 wt % PBI from 10 wt % KOH in ethanol leads to voltaic efficiencies above 58% for all the current densities applied ranging from 0 to 100 A/m^2^. This is due to the introduction of the thin Cl^–^/OH^–^ selective coating that does not alter the reverse bias performance (see supplementary information S8), but does improve the BPM FB performance in the presence of background salts, as shown earlier.

Therefore, coating a thin PBI OH^–^/Cl^–^ selective layer greatly increases the power density of a reverse BPM electrodialysis process that harvests energy from salt-contaminated acids and bases. Moreover, both the discharge power density and the voltaic efficiency are improved in the case of energy storage systems, such as the acid–base flow battery, leading to more energy-efficient processes.

## Conclusion

Background sodium chloride in acid and base greatly increases the bipolar membrane (BPM) overpotential in forward bias due to the accumulation of salt ions at the BPM junction that hinders H^+^ and OH^–^ transport. The ionic blockade phenomenon is especially noticeable when background salt is present in the base compartment due to the larger similarity between OH^–^ and Cl^–^ than between H^+^ and Na^+^, which makes it more challenging to achieve chemoselectivity for the anion couple. As a result, the poor OH^–^/Cl^–^ selectivity of the AEL leads to worse BPM forward bias performance in the presence of a background salt in the base.

Therefore, the BPM’s AEL surface is modified by coating a dense submicrometer-thick poly­(benzimidazole) selective layer from an ethanolic KOH solution. The successful AEL coating with 3 and 4 wt % PBI from 10 wt % KOH in ethanol leads to a reduced sensitivity to the presence of background salt, thanks to the interactions between PBI and FAA-3 that create a dense ionically cross-linked layer at the interface between the two polymers. In addition, the negatively charged deprotonated benzimidazole groups of PBI lead to the Donnan exclusion of Cl^–^ ions, while the OH^–^ ions are still able to hop between the alkaline-doped free volumes of the PBI coating. As a result of the introduced OH^–^/Cl^–^ selectivity, the accumulation of Cl^–^ ions at the BPM junction is reduced, and the BPM FB overpotential is lowered as the mass transport of OH^–^ ions to the junction is no longer hindered by the presence of a Cl^–^ ionic blockade.

A reduced salt sensitivity is highly relevant for applications that retrieve energy from a pH gradient when the acid and base are contaminated with background salt. The BPM peak power density harvested from 0.5 M HCl and NaOH solutions contaminated with 0.1 M NaCl increased from 32 W/m^2^ for an uncoated FBM BPM to 51 W/m^2^ for the best-performing coated BPM. Moreover, the BPM voltaic efficiency at 100 A/m^2^ (dis)­charge was increased from negative values to above 57% for the coated membrane, demonstrating that the presented BPM modification is relevant to systems that harvest energy via BPM reverse electrodialysis.

## Supplementary Material



## Notes

S1: Schematic of the six-compartment setup used for the experiments. S2. SEM images of a Fumatech BPM after forward bias in acid and base and in salt solution. S3: Bode plots supplementary to Figure 3. S4. Schematic of the phenomena at play when casting the PBI solutions in ethanolic KOH on the wet BPM. S5: FT-IR spectra of the Fumatech FBM BPM and FAA-3. S6: Optical and SEM pictures of cast self-standing PBI films. S7: Nyquist plots of the PBI-coated BPMs. S8: Polarization curve of a coated and uncoated FBM BPM in reverse bias (PDF)
