# Supplementary material for: Coated Bipolar Membranes with Improved Forward Bias Performance for Energy Harvesting from Salt-Contaminated Acid and Base
Source: ACS Electrochem. 2025 May 14;1(8):1360–72. doi: 10.1021/acselectrochem.5c00052 (PMC12337089; doi:10.1021/acselectrochem.5c00052)
Supplement: Supplementary file 1 [file ec5c00052_si_001.pdf]

Coated bipolar membranes with improved forward bias performance for energy harvesting from salt-contaminated acid and base

--- Supplementary information ---

Authors: Nadia Boulif, Kitty Nijmeijer, Zandrie Borneman*

*Corresponding author: z.borneman@tue.nl

Affiliation: Membrane Materials and Processes, Department of Chemical Engineering and Chemistry, Eindhoven University of Technology (P.O. Box 513, 5600, MB, Eindhoven, The Netherlands)

Table of content:

S1. Schematic of the six-compartment setup used for the experiments ………..… S2

S2. Images of commercial Fumatech BPM in forward bias between acid and base and in 0.5 M NaCl ……………………………………………………………... S3

S3. Bode plots of the uncoated Fumatech FBM BPM between pure or contaminated acid and base ……………………………………………………….. S4

S4. Schematic of the phenomena at play when casting the PBI solutions in ethanolic KOH on the wet BPM ……………………………………...……. S5

S5. FT-IR of the FAA-3 and the commercial FBM BPM AEL ……………………. S6

S6. Morphology of the coating – additional images ………………………………... S7

S7. Electrochemical Impedance Spectroscopy of coated membrane …………….. S8

S8. Reverse bias of the uncoated and coated BPMs ………………………………. S9

S1. Schematic of the six-compartment setup used for the experiments

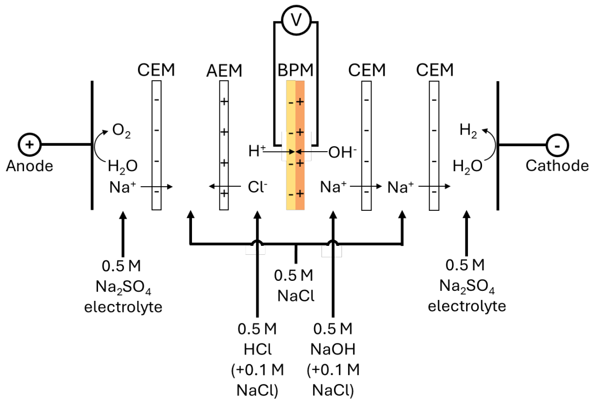

Figure S1. *Schematic of the six-compartment cell used in this study. The CEMs and AEMs were Neosepta CMX-fg and AMX-fg from Astom (Japan). The exposed BPM area was 0.785* cm^2*, while that of the other membranes was 17.3* cm^2*. The 0.5 M* Na_2SO_4 *and 0.5 M NaCl compartments were connected in series. The arrows represent the expected ionic fluxes.*

S2. Images of commercial Fumatech BPM in forward bias between acid and base and in 0.5 M NaCl

SEM pictures (see **Figure S2**) demonstrate that the commercial Fumatech FBM BPM is mechanically stable when put in forward bias between 0.5 M HCl and 0.5 M NaOH, while it delaminates easily when put in forward bias in 0.5 M NaCl. This suggest that the accumulation of salt at the BPM junction leads to the mechanical failure of the BPM due to the high osmotic pressure at the junction as salt ions accumulate.

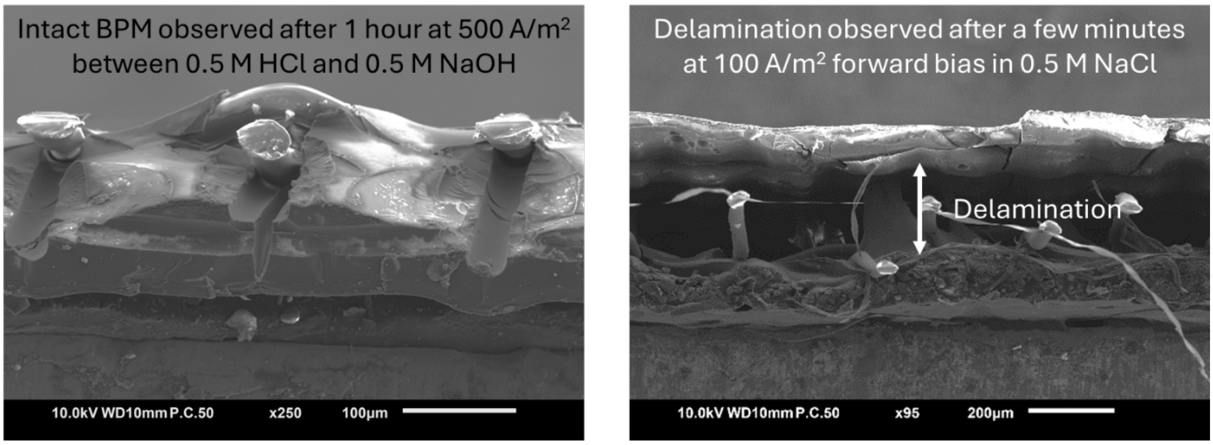

Figure S2. *SEM images of a Fumatech FBM that has been in forward bias between acid and base (left) and in 0.5 M NaCl (right). Clear delamination is observed when the BPM is in forward bias in a salt solution.*

S3. Bode plots of the uncoated Fumatech FBM BPM between pure or contaminated acid and base

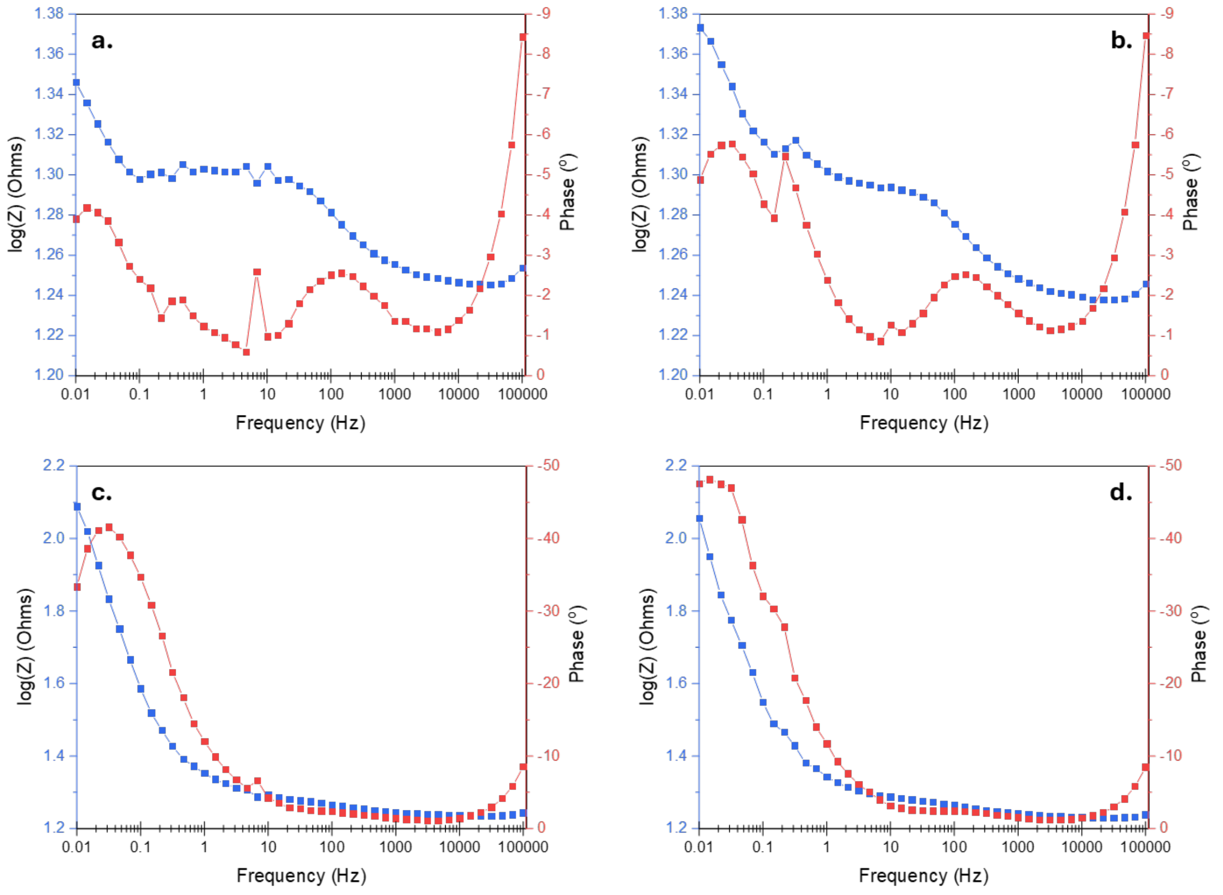

Figure S3. *Bode plots of the uncoated Fumatech FBM BPM at 80 A/m^2 ±8 A/m^2* ***a.*** *between 0.5 M HCl and 0.5 M NaOH,* ***b.*** *with 0.1 M NaCl in the acid* ***c.*** *with 0.1 M NaCl in the base* ***d.*** *with 0.1 M NaCl in the acid and the base.*

S4. Schematic of the phenomena at play when casting the PBI solutions in ethanolic KOH on the wet BPM

The different processes that simultaneously take place when coating the BPM's AEL with the PBI solutions in ethanolic KOH are schematically represented in the following **Figure S4**:

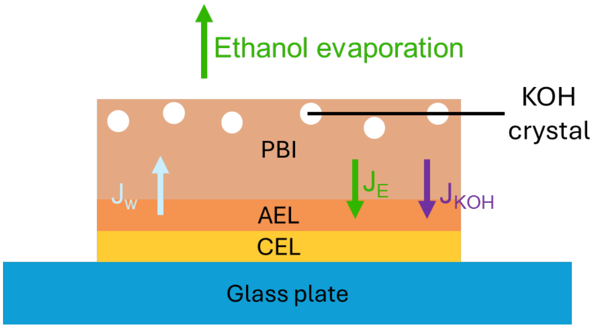

Figure S2. *Schematics of the processes happening during the coating of the PBI solution in ethanolic KOH on a wet commercial FBM. Drawing not to scale.*

S5. FT-IR of the FAA-3 and the commercial FBM BPM AEL

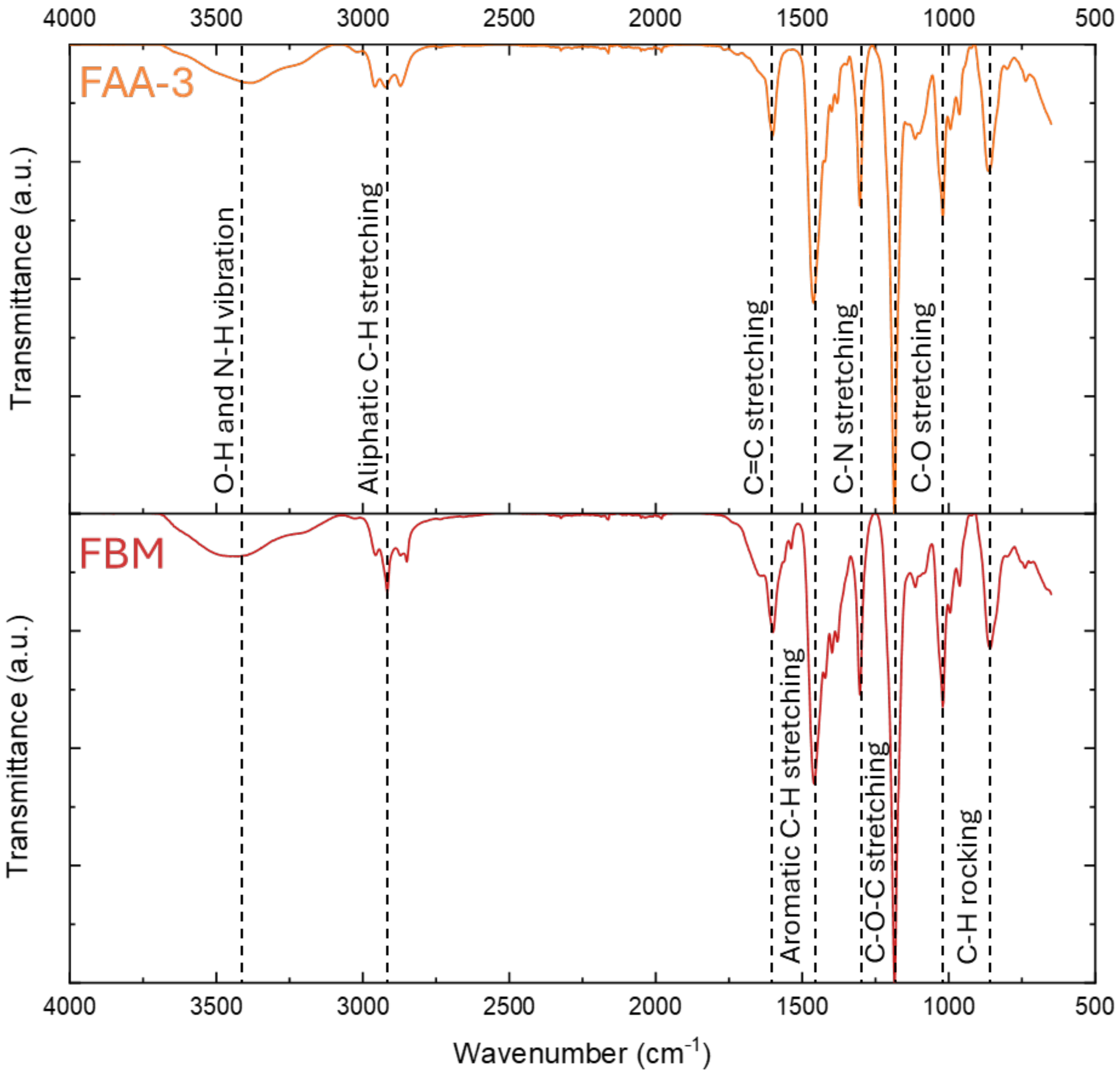

Figure S5. *FT-IR spectra of the FAA-3 AEM and the FBM BPM AEL.*

S6. Morphology of the coating – additional images

- Optical pictures of self-standing cast PBI films

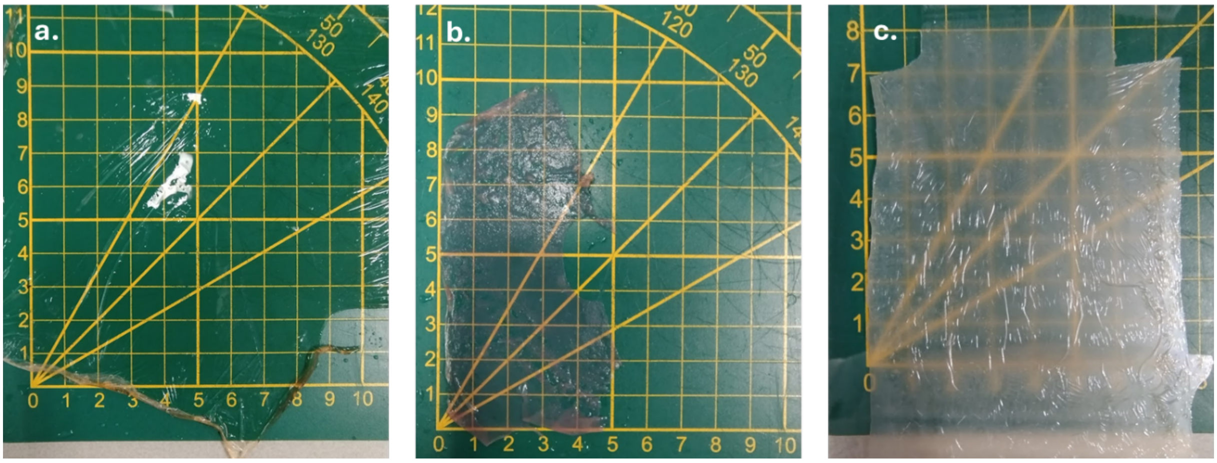

Figure S6. *.Optical pictures of the self-standing* ***a.*** *PBI film cast from a 3 wt% PBI in 10 wt% KOH in ethanol with a 0.20 mm thick casting knife, NIPS in water* ***b.*** *PBI film cast from a 3 wt% PBI in 10 wt% KOH in ethanol with a 0.5 mm thick casting knife, NIPS in water and* ***c.*** *PBI film cast from a 3 wt% PBI in 10 wt% KOH in ethanol with a 0.20 mm thickness, evaporated at room conditions*

- Morphology of membranes under the SEM

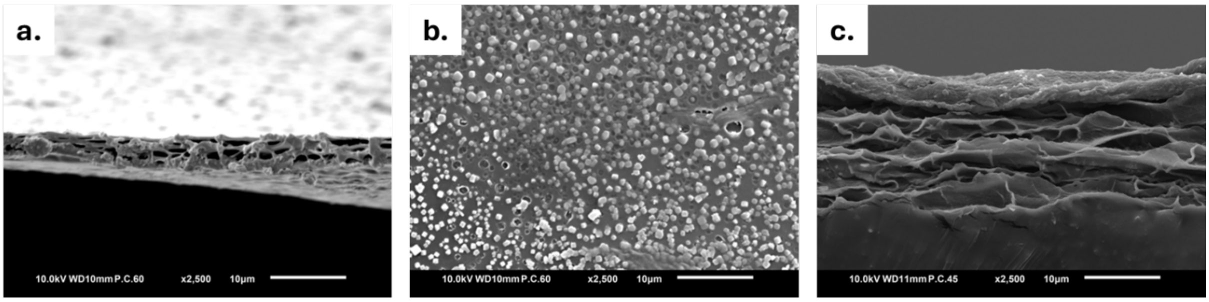

Figure S7*. SEM pictures of the self-standing PBI films* ***a.*** *cross-section of a PBI film cast from a 3 wt% PBI in 10 wt% KOH in ethanol with a 0.20 mm thick casting knife, ethanol evaporated at ambient conditions* ***b.*** *top surface of a PBI film cast from 3 wt% PBI in 10 wt% KOH in ethanol with a 0.50 mm thick casting knife, ethanol evaporated at ambient conditions and* ***c.*** *cross-section of a PBI film cast from 3 wt% PBI in 10 wt% KOH in ethanol with a 0.50 mm thickness, NIPS in water.*

The self-standing film made by evaporation of ethanol has a porous structure due to the KOH crystallization that acts as a pore forming agent. Immersing the cast PBI film directly in water also leads to a porous structure if the cast film is thick (0.5 mm). If the cast film is thin, a dense structure is obtained as a result of the fast ethanol evaporation that densifies the cast film.

S7. Electrochemical Impedance Spectroscopy of coated membranes

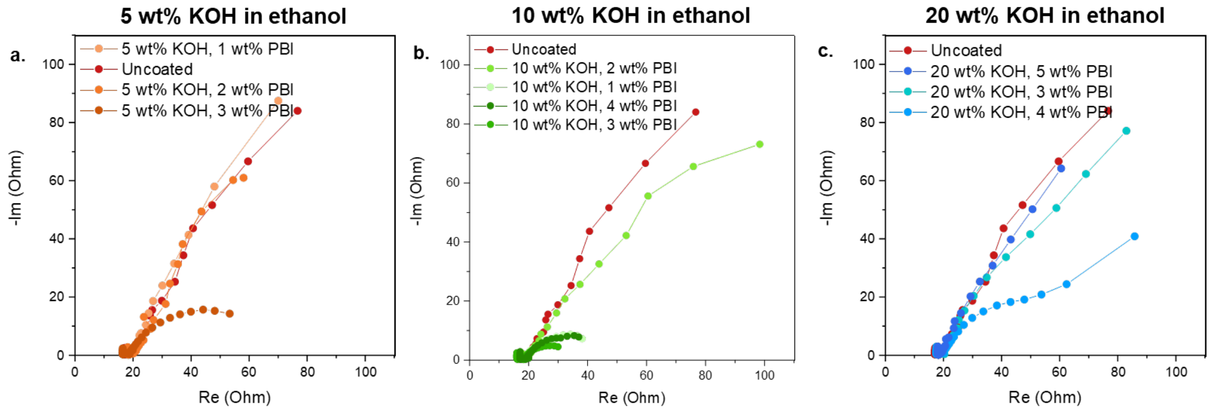

Figure S8. *Nyquist plots of the coated BPMs under a forward bias of 80 A/m*^{2} *with an amplitude of 10%. Results obtained for the* ***a.*** *5 wt% KOH solution,* ***b.*** *10 wt% KOH in solution, and* ***c.*** *20 wt% KOH in solution.*

S8. Reverse bias of the uncoated and coated BPMs

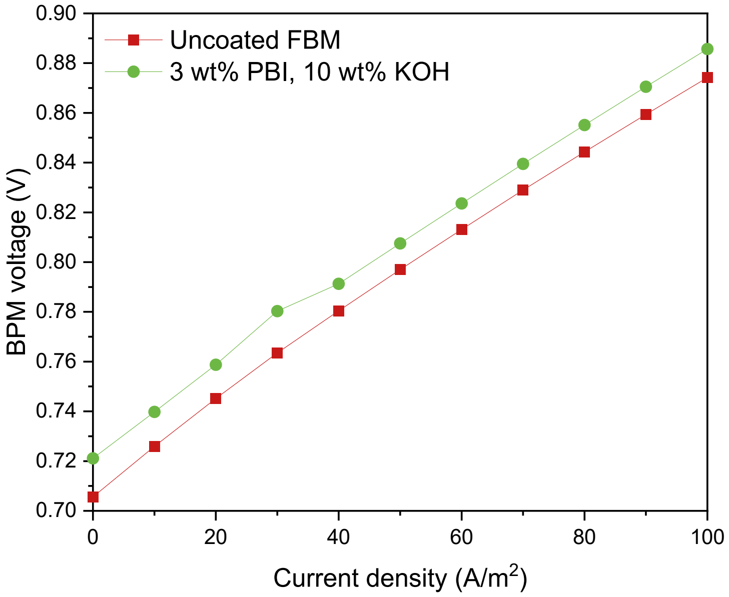

***Figure S9.**. Reverse bias current density-voltage curve of the uncoated and coated FBM (3 wt% PBI, 10 wt% KOH) BPMs used for the long term stability tests.*
